# New Approach Methods to Assess the Enteropathogenic Potential of Strains of the *Bacillus cereus* Group, including *Bacillus thuringiensis*

**DOI:** 10.3390/foods13081140

**Published:** 2024-04-09

**Authors:** Arnaud Fichant, Rachelle Lanceleur, Salma Hachfi, Alexandra Brun-Barale, Anne-Louise Blier, Olivier Firmesse, Armel Gallet, Valérie Fessard, Mathilde Bonis

**Affiliations:** 1 Laboratory for Food Safety, University Paris-Est, French Agency for Food, Environmental and Occupational Health & Safety (ANSES), 94700 Maisons-Alfort, France; arnaud.fichant@anses.fr (A.F.); olivier.firmesse@anses.fr (O.F.); 2 Université Côte d’Azur, CNRS, INRAE, ISA, 06903 Sophia-Antipolis, France; salma.hachfi@ucsf.edu (S.H.); alexandra.brun-barale@inrae.fr (A.B.-B.); armel.gallet@univ-cotedazur.fr (A.G.); 3 Fougères Laboratory, French Agency for Food, Environmental and Occupational Health & Safety (ANSES), 35306 Fougères, France; rachelle.lanceleur@anses.fr (R.L.); anne-louise.blier@anses.fr (A.-L.B.); valerie.fessard@anses.fr (V.F.)

**Keywords:** *Bacillus cereus*, *Bacillus thuringiensis*, enteropathogenicity, Caco-2 cells, *Drosophila melanogaster*, new approach methods

## Abstract

*Bacillus cereus* (Bc) is a wide group of Gram-positive and spore-forming bacteria, known to be the etiological agents of various human infections, primarily food poisoning. The Bc group includes enteropathogenic strains able to germinate in the digestive tract and to produce enterotoxins such as Nhe, Hbl, and CytK. One species of the group, *Bacillus thuringiensis* (Bt), has the unique feature of producing insecticidal crystals during sporulation, making it an important alternative to chemical pesticides to protect crops from insect pest larvae. Nevertheless, several studies have suggested a link between the ingestion of pesticide strains and human cases of food poisoning, calling their safety into question. Consequently, reliable tools for virulence assessment are worth developing to aid decision making in pesticide regulation. Here, we propose complementary approaches based on two biological models, the human intestinal Caco-2 cell line and the insect *Drosophila melanogaster*, to assess and rank the enteric virulence potency of Bt strains in comparison with other Bc group members. Using a dataset of 48 *Bacillus* spp. strains, we showed that some Bc group strains, including Bt, were able to induce cytotoxicity in Caco-2 cells with concomitant release of IL-8 cytokine, a landmark of pro-inflammatory response. In the *D. melanogaster* model, we were able to sort a panel of 39 strains into four different classes of virulence, ranging from no virulence to strong virulence. Importantly, for the most virulent strains, mortality was associated with a loss of intestinal barrier integrity. Interestingly, although strains can share a common toxinotype, they display different degrees of virulence, suggesting the existence of specific mechanisms of virulence expression in vivo in the intestine.

## 1. Introduction

*Bacillus cereus* (Bc) is a highly versatile group of Gram-positive, ubiquitous, spore-forming bacteria able to trigger various types of human illnesses. It encompasses at least eight species defined historically, comprising *Bacillus cereus sensu stricto* (Bc s.s.), *B. thuringiensis* (Bt), *B. anthracis*, *B. mycoides*, *B. pseudomycoides*, *B. weihenstephanensis*, *B. cytotoxicus*, and *B. toyonensis*, while numerous other species have been suggested more recently as new members of the group [1,2]. In the past few decades, several taxonomic revisions have been proposed to classify Bc, for instance, into phylogenetic groups [3], clades [4], or genomospecies [5]. However, the resulting phylogenies do not match completely with historic speciation, and Bc taxonomy remains controversial.

Although Bc can be responsible for sporadic extra-digestive infections such as pneumonia [6], endophthalmitis [7], and septicemia [8], its preferential target remains undoubtedly the digestive tract. In 2021, Bc was reported as the most common causative agent of food-borne outbreaks (FBOs) in France [9], and the leading cause of FBOs due to bacterial toxins in Europe [10]. Interestingly, France was the EU member state reporting by far the highest number of this type of FBO (approximately 90%), probably due to a high level of investigation conducted in this country. This suggests an underestimation of the Bc-associated FBOs cases in the other EU member states.

Depending on the strain, Bc can cause two main types of food poisoning. In the first instance (about 10% of Bc-FBOs), the clinical disorder relies on the ingestion of an emetic toxin—the dodecadepsipeptid cereulide—which is pre-formed in food by vegetative bacteria [11]. Vomiting/nausea-type symptoms then occur rapidly and can be accompanied by multi-organ failure [12] and death in some cases [13]. In the second case (about 90% of Bc-FBOs), the disorder is predominated by diarrheal-type symptoms, although emetic ones are also often reported. Intoxication results from the ingestion of Bc qualified as enteropathogenic, able to germinate and to secrete virulence factors in the small intestine.

Although the complete mechanism of enteropathogenicity of these strains is not fully elucidated, it is accepted to mainly rely on the activity of the pore-forming cytotoxins Hbl [14] and Nhe [15], both tripartite toxins encoded by the *hbl C/D/A* and *nhe A/B/C* genes, respectively, and the single-component β-barrel toxin CytK [16], with the two variants CytK1 and CytK2 encoded by the *cytK1* and *cytK2* genes, respectively. The first one is highly cytotoxic and is restricted to *B. cytotoxicus*. Although unsuccessful, efforts have been made to correlate the presence/absence of these toxins, or their levels of expression and production, with cytotoxicity in some mammalian cell lines [17]. This suggests that Bc’s virulence is restricted neither to the presence nor to the expression of these toxins. De facto, Bc can express numerous other types of factors involved in virulence, such as hemolysins, metalloproteases, phospholipases (sphingomyelinase and PI-PLC in particular), and cell wall peptidases (such as EntFM) [1,18]. Moreover, some of these components act in combination, as shown for instance with Hbl, PI-PLC, and sphingomyelinase in erythrocyte hemolysis [14], or with Nhe and Hbl in inflammatory response [19].

Furthermore, the virulence potency of Bc depends on its fate along the digestive tract, in particular the ability to adhere to the intestinal epithelium [20,21], to swarm [22], and to survive hostile conditions, such as stomach pH. Recent data have demonstrated that, while vegetative cells do not survive in the stomach and duodenum due to acidic pH and the presence of bile salts, respectively, spores can reach the jejunum and the ileum [23,24,25,26]. Meanwhile, a germination step of the spores is necessary before toxin production [27]. Therefore, the prediction and classification of enteropathogenicity of Bc strains is challenging, and new tools to assess the virulence potential of Bc strains need to be developed.

Although the enteropathogenicity of Bc was demonstrated many decades ago, the involvement of Bt (belonging to the Bc group in the broad sense) in food poisoning has been suspected more recently [28]. Bt was described for its entomopathogenic properties at the beginning of the 20th century. During sporulation, the bacteria produce crystals made of δ-endotoxins (Cry/Cyt toxins) [29], which damage the intestinal epithelium of susceptible insect larvae once ingested together with bacterial spores. Spores further germinate in the midgut and proliferate in the *milieu interieur*, leading to the death of targeted larvae by septicemia. This peculiar action led to the development of multiple and effective biopesticides/biocides for the control of specific insect pests, such as Lepidoptera (with Bt ssp. *kurstaki* and *aizawai*, hereafter Btk and Bta) and Coleoptera (Bt ssp. *tenebrionis*, Btt) in farming and forestry, and Diptera (Bt ssp. *israelensis*, Bti) for mosquito control. Bt is now the best-selling biopesticide worldwide [30]. In 2015, it even ranked second among the 30 top-selling insecticides (all types combined), with 32,000 m/t sold (i.e., 17% of the 30 best-selling insecticide sales) [31]. Bt is therefore considered as a valuable alternative to chemical pesticides.

However, Bt is genetically close to the other members of the Bc group, in particular Bc ss, and grouped within the single *Bacillus cereus sensu stricto* genomospecies, according to the taxonomic revision proposed by Carroll et al. in 2020 [5]. As a result, Bt is not routinely discriminated from other Bc, and its involvement in human intoxications is probably underestimated. Some studies have revealed the presence of Bt in food poisoning events [32,33,34]. Up to 20% occurrence was estimated from a large panel of Bc-associated FBOs studied in France in 2021 [35], and the isolated strains could not be distinguished from pesticide strains. Also, Bt carries genes encoding Bc enterotoxins, such as CytK2, Nhe, and Hbl, among others. Nevertheless, the strict implication of Bt in these FBOs cannot be rigorously demonstrated and requires the development of new tools to better characterize their potential enteropathogenicity.

In agreement with the 3R and more recently the 4R principles of animal welfare [36,37], the development of alternative methods is a step forward to assess the putative enteropathogenicity of Bc spores. Therefore, we developed a new methodological approach, based on complementary in vitro and in vivo assays, using human intestinal Caco-2 cells, isolated from a colon adenocarcinoma, and *Drosophila melanogaster* (*D. melanogaster*), respectively. The Caco-2 cell model has been used intensively to study the impact of drugs and other chemicals on the intestine, including transfer across the intestinal barrier [38]. Moreover, the pro-inflammatory IL-8 marker produced by Caco-2 cells was largely used to detect the inflammation potential of a broad panel of chemical and biological agents [39,40]. A few papers report the testing of Bc and Bt extracts on the viability of Caco-2 cells, as well as the adhesion of bacteria to intestinal cells [21,41].

*Drosophila melanogaster* is a well-established model to study host–pathogen interactions due to its amenability, the existence of numerous genetic tools, and the well-conserved genetic networks and signaling pathways regulating intestinal physiology and innate immune response [42,43]. For 15 years, *D. melanogaster* has also been extensively used to decipher the defense mechanisms of the midgut (the functional equivalent of the mammalian small intestine) to combat pathogenic bacteria [44,45]. Importantly, the Bt Cry toxins do not induce acute toxicity in *D. melanogaster* adults [46,47], making it possible to study the virulence of spores and vegetative cells irrespective of the presence of Cry toxins. Our recent work demonstrated that, upon ingestion of a meal contaminated with Bc or Bt spores, spores escape the immune response of the anterior midgut and reach the posterior midgut, where they can germinate to give birth to vegetative cells [25]. Moreover, the germination of Bc or Bt spores in the posterior midgut dampens immune response, fostering their own survival. We also demonstrated that the Bc or Bt behave in the same way in the mouse small intestine [25]. Thus, *D. melanogaster* emerges as an excellent model for studying Bc and Bt virulence in vivo.

In this work, the virulence of 48 *Bacillus* spp. strains was investigated using these two models, after a characterization of the bacterial strains (phylogeny and toxin gene profile). The Caco-2 cells were treated with bacterial supernatants, and *D. melanogaster* was exposed to spores via food. The viability and IL-8 release of intestinal cells as well as the survival rate and intestinal leakiness of *D. melanogaster* were measured.

## 2. Materials and Methods

### 2.1. Bacterial Strains

A panel of 48 *Bacillus* spp. strains was used for this study (Table 1 and Table S1), with 45 Bc strains (including 27 Bt strains) and 3 *Bacillus* spp. non-Bc strains (1 *Bacillus subtilis*, 1 *Bacillus amyloliquefaciens*, and 1 *Bacillus megaterium*). These strains were collected or derived from pesticide products (n = 11), FBOs (n = 13), and collections (n = 24). The mutant SA11∆Cry was obtained from the wild-type (WT) pesticide strain SA-11 (18SBCL487) by a procedure of plasmid curing, as described in [25]. Among the 48 strains, 37 were tested on the Caco-2 model (Subdataset 1), 39 on the *D. melanogaster* model (Subdataset 2), and 28 on both.

### 2.2. Characterization of the Bacterial Dataset

#### 2.2.1. Bt Species Attribution

The attribution of FBO-Bc strains to Bt species was determined by microscopic study of the parasporal crystals under sporulation conditions, as described in [35], according to the standard ISO 7932:2004/Amd 1:2020.

#### 2.2.2. PanC-Typing and Detection of Toxin Genes

Bc strains were classified into phylogenetic groups I to VII according to the panC-typing method originally described in [3]. Briefly, after DNA isolation as previously described [35], a portion of the *panC* gene was amplified with the primer pair (TYGGTTTTGTYCCAACRATGG/CATAATCTACAGTGCCTTTCG) and sequenced by Sanger sequencing (Eurofins Genomics, Ebersberg, Germany), before attribution to groups using the publicly available dedicated classification tool (https://www.tools.symprevius.org/Bcereus/; accessed on 3 April 2024). The presence of *cytk1/2*, *nhe A/B/C*, *hbl C/D/A*, *hlyII*, and *ces* was also detected by conventional PCR, as previously described in [35].

#### 2.2.3. Bacterial Dataset Phylogeny

The genomic DNA of 12 *Bacillus* spp. strains was extracted as previously described [35] and then sequenced using Illumina sequencing technology (2 × 150 bp paired-end sequencing, Bioproject PRJNA1069360, Table S1). Sequencing data obtained from this study (n = 12) and previous ones (n = 22) [35,48] were assembled using a new in-house workflow called Bacflow, as follows. Trimming and quality control of reads were performed using fastp version 0.20.1 (https://github.com/OpenGene/fastp; accessed on 3 April 2024). Intraspecies contamination was performed with ConFindr 0.7.0 (https://github.com/OLC-Bioinformatics/ConFindr; accessed on 3 April 2024) using default parameters, and interspecies contamination was performed with Kraken 2 version 2.1.2 (https://github.com/DerrickWood/kraken2; accessed on 3 April 2024) with a contamination threshold established at 2%. Shovill version 1.1.0 (https://github.com/tseemann/shovill; accessed on 3 April 2024) was used to assemble the genome, and quality control was performed using Mash (https://github.com/marbl/Mash; accessed on 3 April 2024) for the reference search among the NCBI RefSeq genome and quast version 5.0.2 (https://github.com/ablab/quast/releases; accessed on 3 April 2024) for assembly quality and completeness. Assemblies with a coverage depth up to 50X were selected for the rest of the analysis. The assemblies (n = 34) were then associated with 14 genomes from NCBI, to build a k-mer phylogeny using Jolytree v.2.1 [49] with default parameters and annotated using the iTol v.6.8.1 [50] online website.

### 2.3. Bacterial Culture for Supernatant Production

After isolation and amplification on trypticase soy agar-yeast extract (TSA-YE) medium (bioMérieux, Marcy-l’Étoile, France) for 18 h–24 h at 30 °C (except for strains 168, H, and 899, grown at 37 °C), the bacterial strains were pre-cultured for 18 h at 30 °C (or 37 °C for strains 168, H, and 899) in 9 mL of brain–heart infusion (BHI) medium (bioMérieux), seeded from isolated colonies. Nine mL of BHI medium was then inoculated at OD_600nm_ = 0.05 before incubation at 37 °C with shaking (300 rpm) for 5 h. The cultures were then centrifuged (4500× *g*, 4 °C, 20 min), filtered (0.22 µm) to remove residual bacteria, aliquoted, and stored at −20 °C before testing in cytotoxicity assays. Meanwhile, growth curves were performed to ensure that all strains were synchronized at the beginning of the stationary phase.

### 2.4. Spore Production

Bacteria were cultivated on TSA-YE plates (bioMérieux) for 18 h–24 h at 30 °C (except for strains 168, H, and 899, grown at 37 °C) and then transferred by surface-rich inoculation onto hydrolysate of casein tryptone (HCT) agar with 0.3% glucose plates [51] and incubated for 7 days at 30 °C (or 37 °C for strains 168, H, and 899). After checking sporulation using phase-contrast microscopy (i.e., >98% free spores), the bacteria were collected with a swab, resuspended in 20 mL of cold 0.15% NaCl, and heat-treated twice (in liquid solution, then as a bacterial pellet after a 20 min centrifugation at 15,500× *g*) at 70 °C for 20 min to kill the residual vegetative cells. Spores were resuspended once again with 20 mL of cold 0.15% NaCl and then washed twice with cold sterile distilled water (20 and 10 mL, respectively). For the last washing, the suspension was aliquoted by 1 mL, centrifuged for 20 min at 12,900× *g* at 4 °C and frozen at −20 °C overnight. The aliquots were then lyophilized for 24 h with freeze-drying equipment (model RPV2, Serail Tech, Argenteuil, France) before being stored at −20 °C. The colony-forming unit (CFU)/mL of each preparation was determined after serial dilution and numeration on TSA-YE agar plates, from at least two frozen aliquots.

### 2.5. Toxicity Assays on Caco-2 Cells

#### 2.5.1. Cell Line

Caco-2 cells (ATCC, Manassas, VA, USA) were maintained in minimum essential medium containing 5.5 mM D-glucose, Earle’s salts, and 2 mM L-alanyl-glutamine (MEM GlutaMAX™, Gibco, Bromont, QC, Canada) supplemented with 10% fetal bovine serum (FBS, Gibco), 1% non-essential amino acids, 50 U/mL penicillin, and 50 μg/mL streptomycin (Gibco). Cells were cultured at 37 °C in a humidified atmosphere containing 5% CO_2_. Cells were routinely subcultured every 3–4 days and used at passages 29–42 for all experiments.

#### 2.5.2. Cell Treatment

Cells were seeded in 96-well plates at a density of 20,000 cells per well. After 24 h, cells were treated for 24 h with a range of dilutions of bacterial supernatant (0.20, 0.39, 0.78, 1.56, 3.13, 6.25, 12.5, and 25%) in the corresponding medium without FBS. Each concentration was tested in triplicate. A vehicle control (25% BHI) as well as a positive control for IL-8 release (TNF-alpha at 100 ng/mL) were included. At least 3 independent experiments were performed.

#### 2.5.3. Cytotoxicity

Cell viability was assessed using the 3-(4,5-dimethylthiazol-2-yl)-2,5-diphenyltetrazolium bromide (MTT) assay. After the 24 h incubation period, the cell media were collected for IL-8 measurement (see below), and 100 µL of MTT (Sigma-Aldrich, St Louis, MO, USA) solution (5 mg/mL in medium) was added to each well. The plates were incubated at 37 °C for 2 h prior to the solubilization of the formazan crystals with dimethyl sulfoxide (DMSO, VWR, Radnor, PA, USA). The plates were gently shaken for 5 min, and the absorbance was recorded with a FLUOstar Optima microplate reader (BMG Labtech, Ortenberg, Germany) at 570 nm. Three independent experiments and triplicates per experiment were performed. Cytotoxicity was evaluated as cell viability reduction compared to cells exposed to the vehicle (25% BHI) alone, as the highest % BHI that did not affect the viability of Caco-2 cells compared to negative controls. The half inhibition concentration (IC_50_) and standard deviation were calculated for each strain with GraphPad Prism software version 9.5.1, using a Sigmoidal 4PL model.

#### 2.5.4. IL-8 Release

After treatment with the bacterial supernatants, the cell media were collected and frozen at −20 °C until analysis. Release of IL-8 was measured using an enzyme-linked immunosorbent assay (ELISA). Then, 96-well microplates (Nunc maxisorp, Thermo Fisher Scientific, Waltham, MA, USA) were coated with human recombinant IL-8 primary antibodies (Thermo Fisher Scientific) at 1 μg/mL and incubated overnight at 4 °C. Between each step, 3 washes with phosphate-buffered saline (Gibco)-0.05% Tween^®^ 20 (Sigma-Aldrich) were performed. After saturation with SuperBlockTM buffer (Thermo Fisher Scientific) for 1 h, samples and standards (recombinant IL-8, Thermo Fisher Scientific) were added into the wells and incubated at room temperature for 1.5 h. Biotin-conjugated human IL-8 antibodies (Invitrogen, Waltham, MA, USA) at 0.1 μg/mL were then added for 1 h followed by 100 μL of streptavidin-HRP (1:1000, Thermo Fisher Scientific) for 45 min. Finally, 50 μL of the chromogenic substrate 3,3′,5,5′ tetramethylbenzidine (TMB, Thermo Fisher Scientific) was added, and the reaction was stopped with 50 µL of sulfuric acid (2 M, Fluka, Saint-Quentin-Fallavier, France). Plates were read at 405 nm. An IL-8 standard curve enabled us to estimate the concentrations of IL-8 (in pg/mL) in duplicate prior to normalization with the vehicle control, and fold change of IL-8 was calculated. Three independent experiments and triplicates per experiment were performed. An ANOVA was performed with GraphPad Prism software (version 9.5.1), and when significant (*p*-value < 0.05), the mean of each concentration was compared to the vehicle control using Dunnett’s test.

### 2.6. Drosophila melanogaster Rearing and Enterotoxicity Assays

*Drosophila melanogaster* were reared on standardized nutrient medium (0.8% agar, 2.5% sugar, 8% corn flour, 2% yeast) at 25 °C with a 12 h light/12 h dark cycle. For enterotoxicity assays, 5- to 7-day-old WT virgin females (Canton S, stock #64349 obtained from Bloomington Drosophila Stock Center; https://bdsc.indiana.edu accessed on 3 April 2024) were starved for 2 h before being transferred onto a fly vial (10 flies per vial) containing nutrient medium covered with a Whatman filter disk (Grade 401, VWR), soaked with 100 µL of *Bacillus* spp. spore suspension, and incubated at 29 °C. Spore suspensions were obtained by spore dilution in a food dye solution (5% Erioglaucin, Sigma Aldrich); final doses ranged from 10^1^ to 10^8^ CFU/fly, in 1 log increments. A total of 100 µL of food dye or distilled water without spores was used as the negative control. Dead *Drosophila* were counted daily for 15 days and removed as soon as they were found in order to limit contamination of other *Drosophila* due to putative bacterial proliferation in the cadavers. At the same time, events of permeabilization of the digestive epithelium were numerated daily by counting blue *Drosophila* (named smurfs [52]) using a Zeiss Stemi 508 stereomicroscope. For each condition, 10 independent replicates of 10 flies were analyzed. Pictures of *Drosophila* were acquired using a numeric Keyence VHX 2000 microscope. Subsequently, *D. melanogaster* survival data were processed with R (v4.2.2.), using the Survival (v3.5-7) and survminer (v.0.4.9) packages. Survival curves were clustered into 4 virulence groups through a k-mean clustering algorithm, using the clustcurv package (v.2.0.1) [53]. The non-parametric Log-Rank test was performed to compare survival curves.

## 3. Results

### 3.1. Description of the Bacterial Strain Panel

**Figure 1:**
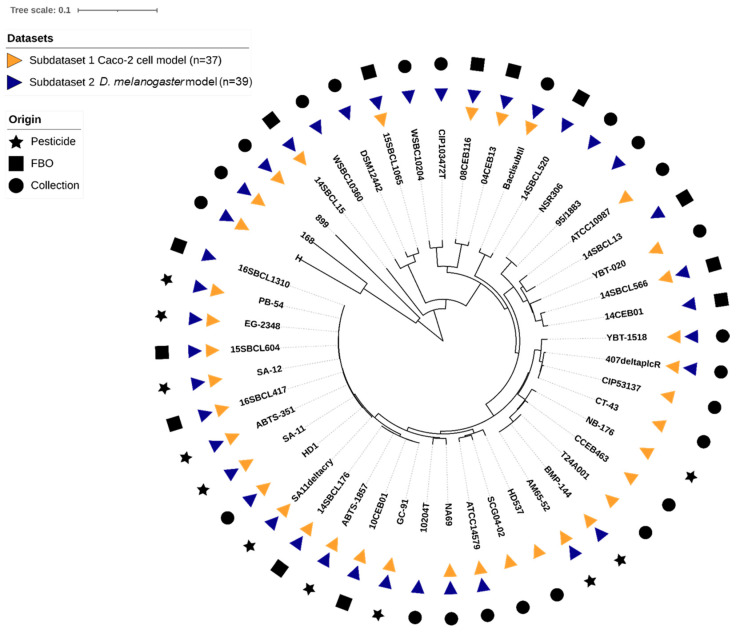
K-mer based phylogeny of the 48 *Bacillus* spp. strains used in this study. The distance-based phylogenetic tree was inferred using JolyTree v.2.1 [49] visualization, and annotation was performed using iTol v6.8.1 [50].

In this study, an overall set of 48 *Bacillus* spp. strains was tested. The set included 27 Bt (11 pesticide strains or mutant, 5 FBO-associated Bt assimilated to pesticide strains [35], and 11 non-pesticide Bt), 18 Bc non-Bt, and 3 *Bacillus* spp. non-Bc (Table 1). Strains belonging to the seven Bc phylogenetic groups (I to VII) were represented (Table S1), with a majority of group IV strains (54%), and a total of 10 Bc species were tested. Using k-mer phylogeny, we confirmed that the genetic diversity of the strains was representative of the Bc group, as compared to the literature (Figure 1). Additionally, we searched for the presence of genes encoding the five major toxins (*cytK1/2*, *nheA/B/C*, *hblC/D/A*, *hlyII*, and *ces*). We concluded that the set included a diversity of toxinogenic profiles, with one major profile: *cytK2*+, *nheA/B/C*+, *hblC/D/A*+, *hlyII*-, *ces*- (31% of the dataset), 10 strains *hlyII*+ (21%), one strain *ces*+ (2%), and one strain *cytk1*+ (2%).

### 3.2. Caco-2 Cytotoxicity of Bacillus spp. Supernatants

**Figure 2:**
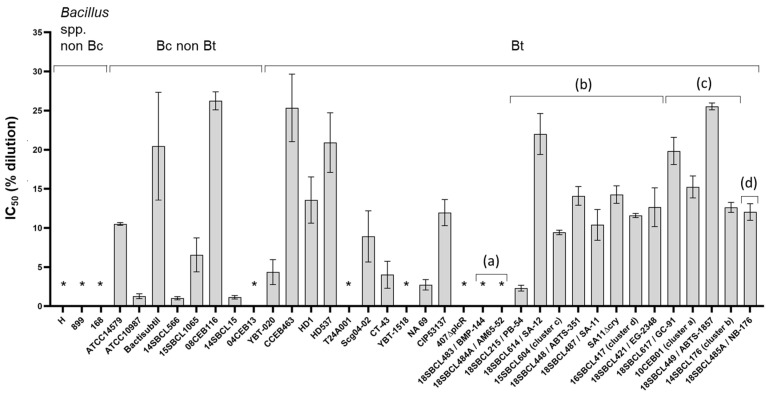
Cytotoxicity of *Bacillus* spp. supernatants on Caco-2 cells. After 24 h of treatment with increasing concentrations of *Bacillus* spp. (n = 37, Subdataset 1) supernatants (from 0.2 to 25% dilution in culture medium), Caco-2 viability was measured using the MTT assay and normalized against the vehicle control, to calculate the half inhibition concentration (IC_50_) for each strain, using the sigmoidal 4PL model (GraphPad Prism software version 9.5.1). Three independent experiments and triplicates per experiment were performed. For each strain, the mean and the standard deviation of the IC_50_ are presented. * Supernatants considered non-cytotoxic (no IC_50_ could be calculated). Bt pesticide strains (or mutant) are annotated (a) to (d), with (a): Bt ssp. *israelensis*, (b): Bt ssp. *kurstaki*, (c): Bt ssp. *aizawai*, and (d): Bt ssp. *tenebrionis*.

First, the toxicity of 37 *Bacillus* spp. supernatants (Subdataset 1) was assessed on intestinal Caco-2 cells after a 24 h treatment (Figure 2 and Figure S1, Table S2). Among the 10 strains of Bt pesticides tested, only the two Bt ssp. *israelensis* (Bti) strains did not induce any cytotoxicity. Moreover, seven non-pesticide strains (three *Bacillus* spp. non-Bc strains and four Bc/Bt strains) did not induce any cytotoxicity. In contrast, all the pesticide and FBO-Bt strains from ssp. *kurstaki* and *aizawai* (n = 11) showed an intermediate level of cytotoxicity (IC_50_ ranged from 2.3 to 25.5%). As expected, the mutant SA11∆cry and WT strain SA-11 showed a similar IC_50_ (14.5 and 10.1%, respectively), while no cytotoxicity was observed with the mutant 407∆plcR. Among the other tested strains, different levels of cytotoxicity were observed. The most cytotoxic strains were a *B. cytotoxicus* strain (14SBCL15) and a *B. paranthracis* strain (14SBCL566), with an IC_50_ around 1.0%. In contrast, the less cytotoxic strain was a *B. wiedmannii* strain (08CEB116), with an IC_50_ of 26.2%.

### 3.3. Pro-Inflammatory Effect of Bacillus spp. Supernatants

**Figure 3:**
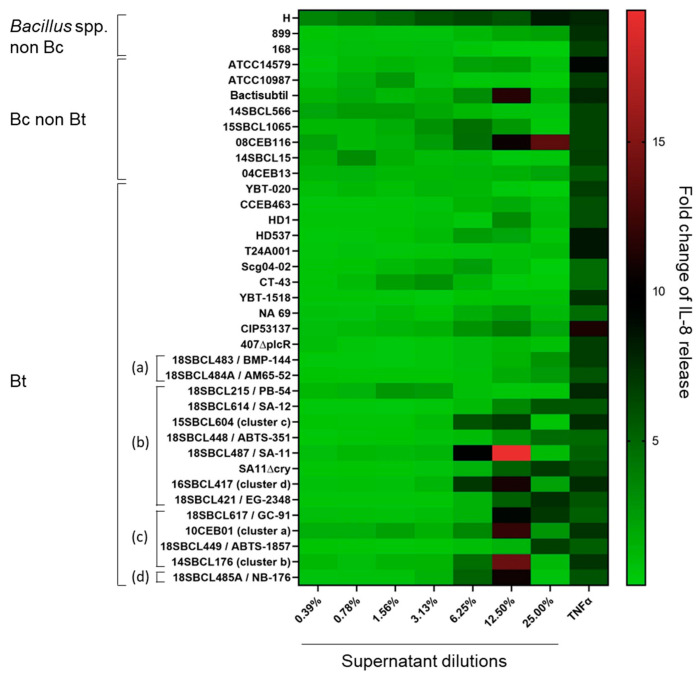
Heatmap of IL-8 release induced by *Bacillus* spp. supernatants on Caco-2 cells. After 24 h of Caco-2 cell treatment with *Bacillus* spp. supernatants (n = 37, Subdataset 1), the amount of IL-8 released into the culture medium was quantified by ELISA and normalized against the vehicle control to calculate the fold-changes of IL-8 release. Bt pesticide strains (or mutant) are annotated (a) to (d), with (a): Bt ssp. *israelensis*, (b): Bt ssp. *kurstaki*, (c): Bt ssp. *aizawai*, and (d): Bt ssp. *tenebrionis*.

In parallel to the MTT tests, the release of IL-8 was measured in Caco-2 cell media (Figure 3 and Figure S1, Table S2). The results showed no significant increase in IL-8 release compared to the vehicle control for more than half of the strains (20/37), including two *Bacillus* spp. non-Bc, five Bc, ten Bt non-pesticide, and three Bt pesticide (two Bti and one Btk). However, for the rest of the strains (n = 17), IL-8 release significantly increased (*p*-value < 0.05), in particular at the two highest tested concentrations (12.5 and 25%) of supernatant. These strains included all the Bt pesticide strains ssp. *aizawai*, *tenebrionis*, and *kurstaki*, except one (the Bt pesticide PB-54) (n = 6), one Bt non-pesticide, three Bc non-Bt, and the *Bacillus amyloliquefaciens* H. The maximum IL-8 fold-change (19.4) was obtained with the Bt pesticide 18SBCL487/SA-11. Interestingly, except for strain H, the increase in IL-8 release was concomitant to a strong decrease in cell viability (Figure S1).

### 3.4. Effect of the Ingestion of Bacillus spp. Spores on D. melanogaster Lethality

**Figure 4:**
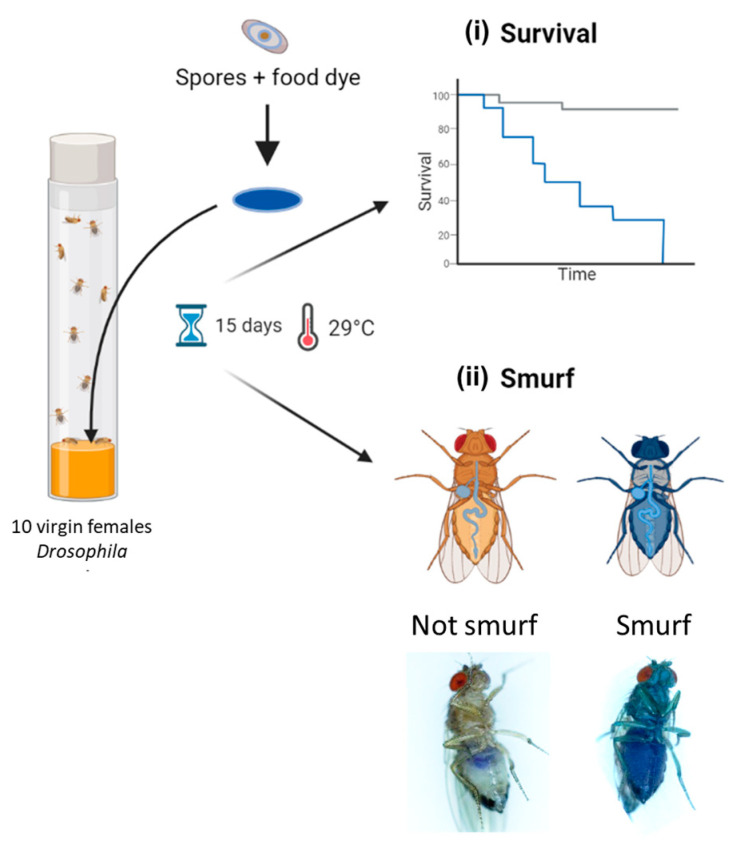
Experimental design of *Drosophila melanogaster* enterotoxicity assays. Flies were exposed to *Bacillus* spp. spores deposited on the surface of the nutritive medium via a Whatman filter disc soaked in spores resuspended in a blue dye solution. Flies were observed daily for 15 days to count (**i**) lethal events and (**ii**) permeabilization events of the digestive epithelium, leading to blue flies (i.e., Smurfs). Parts of this diagram were designed using BioRender (https://www.biorender.com/; accessed on 3 April 2024).

**Figure 5:**
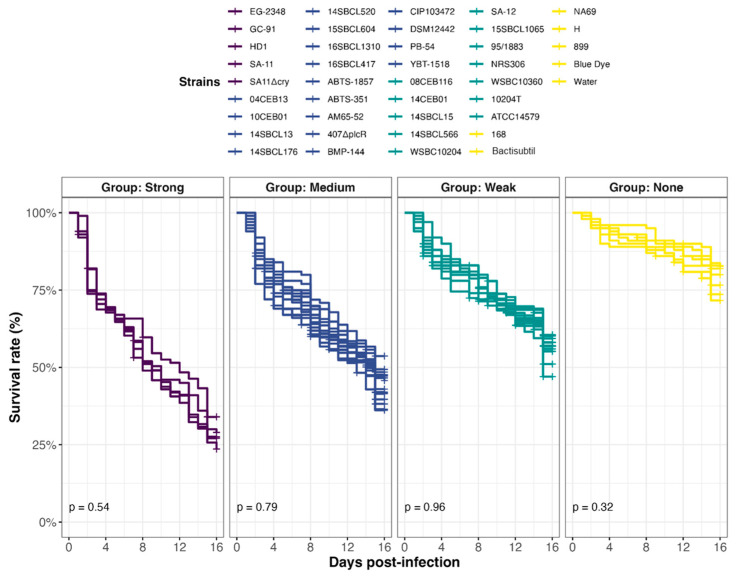
Effect of *Bacillus* spp. spore ingestion on *Drosophila melanogaster* survival. Flies were exposed to 10^7^ CFU/fly of *Bacillus* spp. spores (n = 39, Subdataset 2), or water or blue dye as negative controls, via food and incubated at 29 °C. Lethality was followed up for 15 days (10 independent replicates of 10 flies per condition), and the survival rates were calculated. Strains were clustered into 4 virulence groups (Strong, Medium, Weak, and None) using a k-mean algorithm (clustcurv v.2.0.1), and *p*-values from intra-group curve comparisons were calculated with Log-rank tests (R Survival package v3.5-7).

In all, 39 *Bacillus* spp. strains were tested in an in vivo toxicity assay using *D. melanogaster* (Figure 4). Because fly exposure to a dose of 10^6^ Bc spores per fly did not induce lethality under conventional rearing conditions (i.e., 25 °C) [25,47], we chose a dose of 10^7^ spores/fly and reared them at 29 °C. Under this condition, flies were more sensitive to infection, enabling to discriminate different levels of virulence between pathogens. Four virulence groups were obtained by clustering (Figure 5, Table S2): Strong (n = 5 strains), Medium (n = 17 strains), Weak (n = 12 strains), and None (n = 5 strains), characterized by a median survival rate of 28%, 46%, 57%, and 77%, respectively, at 15 days post-infection. First, the three *Bacillus* spp. non-Bc tested did not promote *D. melanogaster* mortality compared to negative controls (None toxicity group). Regarding the Bc non-Bt panel (n = 17), the tested strains were dispatched into three groups: Weak (11/17 strains), Medium (5/17 strains), and None (1/17 strains). Finally, the Bt strains were mainly categorized into the Strong and Medium groups. Of note, 90% (9/10) of the pesticide strains clustered into these two groups, and 100% (5/5) of the FBO-Bt “pesticide-like” were assigned to the Medium group. Therefore, it appears that the stressful conditions of infection used during our study increased the sensitivity of flies to the enteropathogenicity of those Bt strains. Interestingly, the pesticide strain SA-11 and its mutant SA11∆cry were both classified into the Strong group, suggesting that the crystals of Cry toxins were not the cause of mortality.

### 3.5. Early Mortality of D. melanogaster Correlates with Intestinal Leakiness

**Figure 6:**
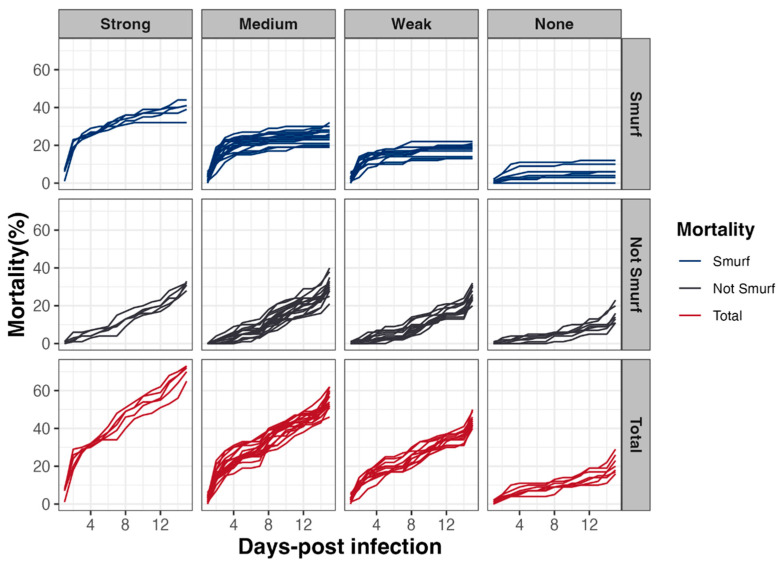
*Drosophila melanogaster* mortality associated with Smurf events. In parallel to lethality monitoring, intestinal barrier breakdown events (i.e., blue flies or Smurfs) using a blue dye were counted, to estimate the relative proportion of fly lethality associated or not with intestinal barrier breakdown, as a function of time and according to the 4 virulence groups previously established.

To address the question of the mechanisms involved in mortality induction, a Smurf assay in parallel to survival tests was carried out. This assay relies on the use of a blue food dye, in which the *Bacillus* spp. spores are resuspended and which turns flies blue as soon as their digestive epithelium is permeabilized, allowing the dye, along with spores, to diffuse in the whole body (Figure 4). Our analyses revealed that, irrespective of the virulence group, early mortality was mainly associated with a rupture of the intestinal barrier (i.e., occurrence of Smurf events in the first 48 h following infection) (Figure 6). Interestingly, the Smurf type represented a mean of 56 ± 3% and 30 ± 12% of the total dead flies in the Strong and None groups, respectively. Altogether, our data showed that the more virulent strains induced early lethality associated with a loss of intestinal barrier, suggesting that flies died by septicemia.

### 3.6. Drosophila melanogaster Exposure–Response Relationship

**Figure 7:**
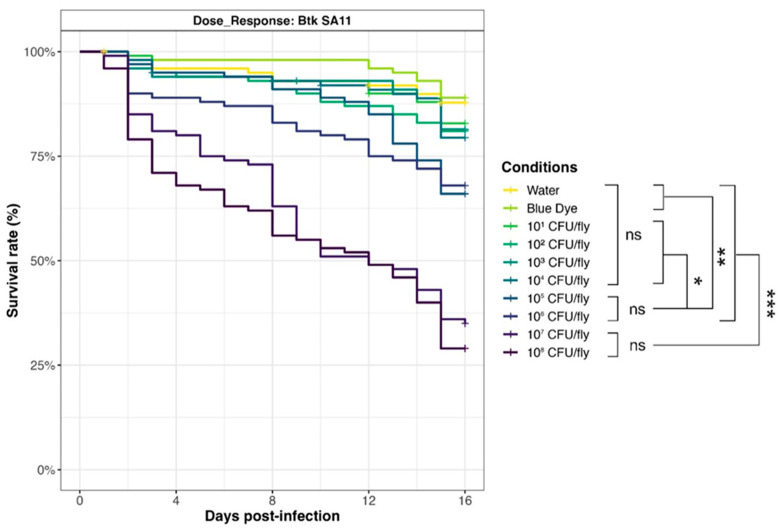
Dose–response of *Drosophila melanogaster* exposed to Btk SA-11 spores. Flies were exposed from 10^1^ to 10^8^ CFU/fly, or water or blue dye as negative controls, via food and incubated at 29 °C. Lethality was monitored for 15 days (10 independent replicates of 10 flies per condition), and the survival rates were calculated. * *p*-value < 0.05, ** *p*-value < 0.005, *** *p*-value < 0.0005, and ns = non-significant, Log-Rank test (R Survival package v3.5-7).

To evaluate the relationship between *D. melanogaster* mortality and *Bacillus* spp. spore exposure, we selected a Bt pesticide strain from the Strong virulence group (SA-11) and carried out survival assays with an increasing amount of spores (from 10^1^ to 10^8^ CFU/fly, in 1 log increments) (Figure 7). Results showed that survival curves for doses of 10^7^ and 10^8^ CFU/fly were not significantly different, pointing out that the maximum effect (around 30% survival rate) was achieved in the tested conditions. Mortality decreased drastically for 10^6^ and 10^5^ CFU/fly (70% survival rate), but rates were still significantly different from 10^4^ CFU/fly, or below (around 80% survival rate), and compared to the negative controls with blue dye and water (90% survival rate).

### 3.7. Comparison of Toxicity Results from the Two Experimental Models

Within the dataset generated in this study, 28 bacterial strains were assessed for their toxic potential on both Caco-2 cells and *D. melanogaster*. The comparison revealed that the results were consistent for 21/28 strains (75%): toxicity was detected in the two models (i.e., from Weak to Strong in *D. melanogaster* and IC_50_ ≤ 26.2% in Caco-2 cells, Table S3). In contrast, for 7/28 strains (25%), the toxic response on the two models diverged, suggesting different virulence mechanisms and specificities in vivo and in cell culture. Interestingly, among the 21 strains found to be toxic in both models, 67% (n = 14/21) were associated with the *cytK2*, *nhe*+, *hbl*+, *hlyII*-, and *ces*- toxinogenic profile, whereas this profile was found in 31% of the overall dataset. Also, among the five strains *cytk2*+, *nhe*+, and *hbl*+, which gave opposite results between the two models, four carried the *hlyII* gene, and were toxic in *D. melanogaster* but not on Caco-2 cells. This suggests that HlyII could be specifically involved in *D. melanogaster* toxicity.

## 4. Discussion

In this study, we first assessed the enterotoxic potential of a panel of *Bacillus* spp. strains by treating human intestinal cells (Caco-2 cell line) with bacterial culture supernatants. The results showed that a broad range of cytotoxicity values could be observed, in line with the literature [54,55,56,57]. *Bacillus paranthracis* (14SBCL566) and *B. cytotoxicus* (14SBCL15) induced the highest levels of cytotoxicity, while the non-cytotoxic strains included one “environmental” Bt, a *B. weihenstephanensis* (species assumed to be of low virulence [58]) and three non-Bc *Bacillus* spp., including *B. amyloliquefaciens*, classified as a “Qualified presumption of safety” organism by the European Food Safety Authority (EFSA) [59].

Additionally, our results showed that the supernatants of Bt pesticide strains from ssp. *kurstaki* and *aizawai* displayed an intermediate level of cytotoxicity among the panel of strains tested. This outcome argues in favor of a possible link between Bta/Btk pesticides and FBOs, as previously described [35]. Although one study classified a few Bt pesticide strains (SA-11, ABTS-351, and ABTS-1857) as “medium-level enterotoxin producers” based on Vero cell cytotoxicity [33], Bt species have been poorly studied for their enterotoxic potential so far, contrary to other Bc strains. This is the first time to our knowledge that all the pesticide strains approved in Europe were assessed on an intestinal human cell model and compared to a large panel of *Bacillus* spp. strains.

The enterotoxicity of Bc strains can result from the activity of multiple virulence factors, in particular enterotoxins triggering deleterious effects on mammalian cells and tissues [1]. However, the exact underlying mechanisms have not been fully elucidated, probably due to (i) the existence of combined toxin effects and (ii) the intrinsic specificities of the strains or species (e.g., existence of toxin variants or transcriptional/translational regulations). This probably explains why the virulence phenotypes observed in this study were not associated with specific toxigenic signatures (regarding the toxins identified, Table S1) and fully justifies this new experimental approach, which does not focus only on a single virulence gene.

In this study, we showed that, over a broad range of *Bacillus* spp. strains, the culture supernatants of some strains could induce cytotoxicity on intestinal Caco-2 cells. Other studies have already used this cell line to detect the virulence of *Bacillus* strains. The cytotoxicity of Caco-2 cells was observed when some components dependent on the PlcR operon were secreted in the bacterial culture supernatants [21]. Consistently, we did not show any cytotoxicity with the *plcR* mutant. The bacterial control strain ATCC14579 was tested in another study on differentiated Caco-2 cells [60]. The authors showed that the supernatant was highly cytotoxic after 3 h of treatment. Although this is difficult to compare with our results due to differences in the experimental design, we noticed that this strain was also cytotoxic in our study, with an IC_50_ of 10.5. The combined effect of Hbl and Nhe on Caco-2 cytotoxicity has been outlined in several papers [17,56,61]. However, due to the low number of Bc strains not producing both toxins in our study, we are unable to confirm this combined effect. Cytotoxicity on Caco-2 has been also investigated with a panel of 24 Bt strains isolated from foodstuffs, animals, and soil, as well as biopesticides, by Schwenk et al. [41]. Except for one, they observed that all the pesticide strains tested showed cytotoxicity, with a classification of moderately to highly cytotoxic based on the amount of Hbl and Nhe toxins produced. Unfortunately, the comparison between the IC_50_ values they obtained and the ones we calculated is difficult, as the expression units differ as well as the experimental design. In contrast to our study, no strain of Bt *israelensis* was tested by Schwenk et al. However, Hansen et al. reported that Bt ssp. *israelensis* and *aizawai* strains had low pathogenic potential, considering various effects, including some toxic measurements on Caco-2 cells, while Bt ssp. *tenebrionis* and *kurstaki* strains had a pathogenic potential similar to the pathogenic strains tested [62]. Parasporins, belonging to the Cry proteins, have been shown to affect the viability of Caco-2 cells as well as other mammalian cell lines [63,64]. However, as for the other Cry toxins, they are produced during sporulation, and as protoxins, requiring activation by insect midgut proteases or experimentally by enzymes such as proteinase K [65]. Therefore, it is unlikely they were involved in the cytotoxicity observed in the present study, in agreement with the results obtained with our mutant ∆cry.

Interestingly, it is the first time that the IL-8 pro-inflammatory marker was measured after Caco-2 treatment with *Bacillus* supernatants. Our study brought to light that Caco-2 cytotoxicity was associated with IL-8 release for a few strains, in particular Bta/Btk. IL-8 is a CXC-family chemokine involved in immune cell recruitment to fight against allochthonous bacteria through induction of the tumor necrosis factor alpha (TNF-alpha) [66]. Recently, Buchacher et al. revealed that Bc could produce extracellular vesicles, mostly smaller than 0.2 µm, in culture [54]. Therefore, these vesicles, present in the bacterial supernatants, can be a pathway to deliver Nhe, sphingomyelinase, and PLC to Caco-2 cells. Such vesicles can also induce TNF-alpha in a few hours in human primary monocytes infected with other bacteria [67,68]. Hence, we can assume that these vesicles could be involved in IL-8 release, though the signaling bacterial compound remains to be identified. Upon ingestion of spores, Bt can persist for several days in the digestive tract of mammals [25,69,70,71], and vegetative cells can induce inflammatory responses in the *Drosophila* intestine [72]. It cannot therefore be ruled out that the repeated exposure of humans to Bt via food [73,74] may trigger a chronic inflammatory reaction in the digestive tract, ultimately leading to chronic inflammatory diseases in which TNF-α plays a key role [75].

To further investigate the enteropathogenic potential of Bc, we developed an in vivo toxicity assay, using the insect model *D. melanogaster*. De facto, this latter constitutes a model of choice, because of the high level of conservation of the innate immune response and the intestinal physiology between *Drosophila* and vertebrates [42,44,76]. Thus, oral infection tests allowed us to classify the studied strains into four degrees of virulence (ranging from the absence of mortality induction to the induction of a “strong” mortality).

Virulence assessment of pathogens requires us to consider both the pathogen and the host. The use of cell cultures can provide informative results to identify some key virulent factors and their underlying mechanisms of action. However, cell monoculture does not mimic the different cell types and their associated functions compared to in vivo models. For instance, the intestine is composed of many cell types (enterocytes, enteroendocrine cells, Paneth cells, progenitors, stem cells, etc.) that communicate to coordinate defenses against pathogens. Therefore, the use of Caco-2 cells cannot illustrate the full intestinal physiology. In fact, more complex intestinal cell systems as well as in vivo models can integrate the response of different cell types and tissues. However, mammalian in vivo models such as rats or mice do not comply with ethics requirements. In this study aiming to develop a new approach method, we chose to assess the enteropathogenic potential of Bc strains in *D. melanogaster*, based on our previous work [25,72,77] and in light of its well-conserved intestinal physiology and innate immune response compared to mammals [78,79].

Our data showed that exposing a stressed fly to 10^7^ spores (by incubation at 29 °C, which facilitates the detection of different virulence phenotypes) via the diet makes it possible to clearly discriminate four groups of virulence. Noteworthy is that this exposure dose must be balanced by the fact that all spores present in the diet will not be ingested. The bacterial groups distinguished by a different phenotype can be further explored to characterize their mechanisms of virulence, such as the rate of germination and proliferation, enterotoxin production, gut lining adhesion, and resistance to host anti-microbial peptides. Importantly, such stressed insects can be considered as a model of a weakened population, more sensitive to opportunistic infections.

The tested dose of 10^7^ CFU/fly is about 20 times the Bt pesticide dose found on fruits/vegetables after treatment at the manufacturer’s maximum recommended dose of ~1 × 10^14^ CFU/ha [80] (calculation carried out by referring to the exposure area, considering for the *D. melanogaster* assay the use of a 5 cm^2^ Whatman filter disk). Such a dose will likely never be reached in the field, but our approach allows us to pinpoint the opportunistic pathogenicity (though non-lethal) of a given strain and to unravel the underlying mechanisms. Our results revealed that, at this dose, 90% of the Bt *kurstaki* and *aizawai* pesticide strains are grouped into the Strong and Medium virulence groups. This mortality cannot be attributed to Cry toxins, since the Btk∆Cry mutant belongs to the group of Strong virulence as well. In addition, the use of a food coloring agent outlined that the mortality observed during the first days is associated with a loss of integrity of the intestinal epithelium, allowing the diffusion of bacteria in the *milieu interieur* and their proliferation, likely causing sepsis. Hence, our data suggest that, at lower doses, Bta/Btk strains may be harmful for the intestinal epithelium (e.g., by inducing diarrheal symptoms), although fortunately not leading to death.

Interestingly, the Bt mutant strains SA11∆cry and 407∆plcR (PlcR being the transcriptional regulator of many extracellular Bc virulence factors, including enterotoxins [31]) displayed a similar virulence profile, in terms of mortality events and intestinal permeabilization, in *D. melanogaster* compared to their WT counterparts. These observations pointed out that virulence could be related to other mechanisms than the action of insecticide crystals (produced jointly with spores) and the production of enterotoxins.

The *D. melanogaster* model brings real added value for predicting the virulence potential of Bc enteropathogenicity. Clearly, it allows for the study of spores instead of vegetative cells and therefore takes into account the ability of spores to resist stomach acidic pH and then to germinate in situ along the digestive tract, an essential step for virulence factor production and pathogenicity expression. For example, the probiotic strain “Bactisubtil” induced a cytotoxic effect on Caco-2 cells, while no mortality was observed in *D. melanogaster*. This discrepancy may be explained by the fact that the germination capacity of this Bc strain can be impaired in the intestine, as shown in vitro in [56]. In fact, other strains, among the panel tested in this study, revealed divergent virulence profiles between the two models, suggesting that mechanisms such as germination efficiency, enterotoxin regulation, bacterial adhesion or mobility, and model physiology could be involved and would be worth investigating. This illustrates the strength of this study in combining two complementary models.

## 5. Conclusions

In this study, we assessed and compared the enteropathogenic potential of a panel of different Bt strains used as biopesticides. We proposed to investigate toxicity on two models, intestinal Caco-2 cells, treated with bacterial supernatants, and the insect *D. melanogaster*, exposed to spores via food. Moreover, we compared the results obtained with Bt biopesticides with a large range of Bc species in order to look for any specific outcomes. As expected, our results exhibited a range of responses in each model over the panel of Bc strains tested.

Therefore, the enterotoxic potential of Bt strains used as pesticides ranged from an absence of effect to a deleterious outcome, depending on the endpoint and model used. Our findings, showing toxicity for some Bt biopesticides, are consistent with the incrimination of Bt pesticide-like bacteria in food poisoning events. However, these results should be put in balance with the effectiveness of Bt pesticides and the possibility they offer to reduce the use of controversial chemical pesticides.

Although the secreted factors, including pore-forming enterotoxins, are undoubtedly implied in toxicity, other crucial factors are also involved. Some strains showed virulence in *D. melanogaster*, although their supernatants were not cytotoxic in Caco-2 cells, and vice versa, underlying the complementarity of both experimental approaches. De facto, as an in vivo model, *D. melanogaster* also reflects the capacity of Bc spores to overcome various organism defenses, aimed at countering allochthonous vegetative bacteria.

Collectively, this work offers new perspectives for predicting the virulence potential of enteropathogenic Bc, including Bt pesticides, and for the study of the associated mechanisms, in order to promote management rules for safe use.

## Figures and Tables

**Table 1:** Distribution of the 48 strains of *Bacillus* spp. used in this study. More details are given in Table S1.

Global Dataset (n = 48)		Subdataset 1 (Caco-2 Cell Model)	Subdataset 2 (*D. melanogaster* Model)
Bc non-Bt (n = 18)	8	17	
Bt (n = 27)	Bt pesticide (or mutant)	11	10
	FBO-Bt “pesticide-like” *	4	5
	Bt non-pesticide (or mutant)	11	4
*Bacillus* spp. non-Bc (n = 3)		3	3
Total		37	39

Abbreviations: Bc = *Bacillus cereus*; Bt = *Bacillus thuringiensis*; FBO = Foodborne outbreak; *D. melanogaster* = *Drosophila melanogaster*. * Strains found undistinguishable from pesticide strains in [35].

## Data Availability

Data is contained within the article or Supplementary Material. The sequencing data used in this study are available in NCBI databases.

## Supplementary Materials

The following supporting information can be downloaded at: https://www.mdpi.com/article/10.3390/foods13081140/s1. Table S1. Description of the bacterial dataset (n = 48) used for the study. Abbreviation: nd = not detected, N/A = not applicable, FBO = Foodborne outbreak, BGSC = Bacillus Genetic Stock Center (https://bgsc.org/ accessed on 3 April 2024). ^a^ Species assignments were compiled from [35], NCBI, BGSC, or defined using BTyper3 (https://github.com/lmc297/BTyper3 accessed on 3 April 2024) for previously undescribed strains. ^b^ Subspecies metadata were collected from [35] or NCBI. ^c^ panC group attribution of Bc strains was performed with (https://www.tools.symprevius.org/Bcereus/ accessed on 3 April 2024) from partial *panC* gene sequencing. nd was indicated in cases of unsuccessful *panC* amplification. * Refers to genomic clusters a to d, previously defined in [35]. Table S2. Compilation of results obtained in this study for each of the 48 strains of *Bacillus* spp., with Caco-2 cell and *D. melanogaster* models. Abbreviations: N/A = not applicable, IC_50_ = half inhibition concentration. Table S3. Comparison of strains profiles assessed with both models Caco-2 cell and *D. melanogaster* (n = 28). Strains associated with an Caco-2 cell IC_50_ < 26.2 and a *D. melanogaster* virulence group Low, Medium, or High are considered to give consistent results between the two models (n = 21, highlighted in green). The others (n = 7) are highlighted in orange. Abbreviations: nd = not detected, N/A = not applicable, IC_50_ = half inhibition concentration. Figure S1. Effect of *Bacillus* spp. supernatants on Caco-2 cell viability and IL-8 release after a 24 h treatment. The % cell viability and fold-change of IL-8 release compared to cells exposed to the vehicle alone are presented. Three independent experiments and triplicates per experiment were performed. For IL-8, statistical analyses were performed by ANOVA and Dunnett’s test. * *p*-value < 0.05, ** *p*-value < 0.01, *** *p*-value < 0.001, **** *p*-value < 0.0001.
