# Supplementary figures and images for: New Approach Methods to Assess the Enteropathogenic Potential of Strains of the Bacillus cereus Group, including Bacillus thuringiensis
Source: Foods. 2024 Apr 9;13(8):1140. doi: 10.3390/foods13081140 (PMC11048917; doi:10.3390/foods13081140)

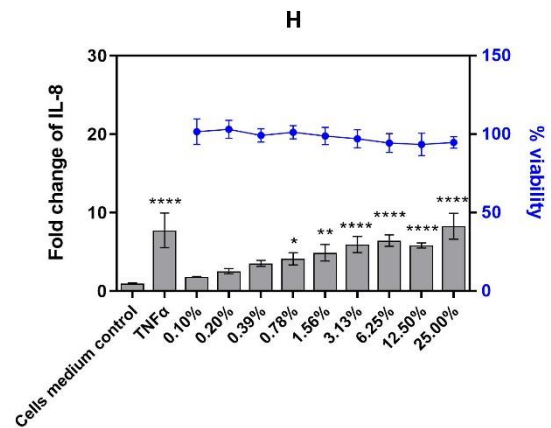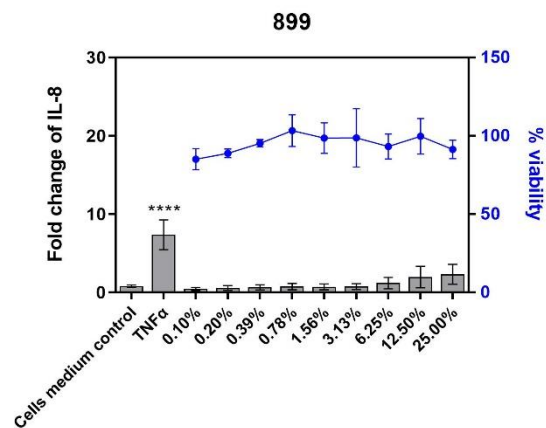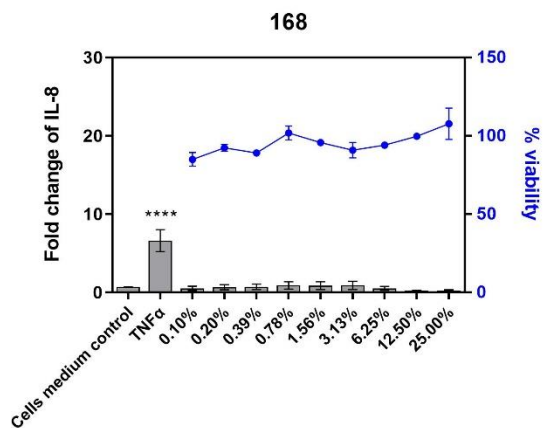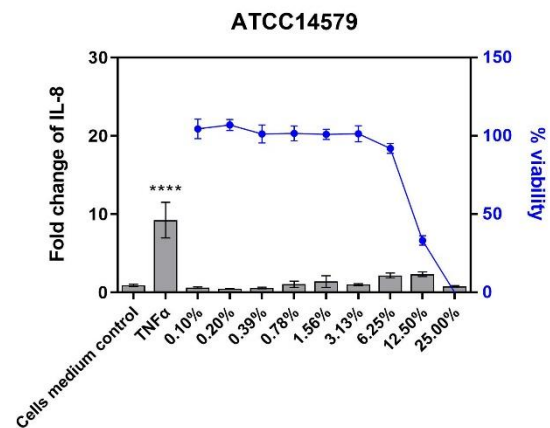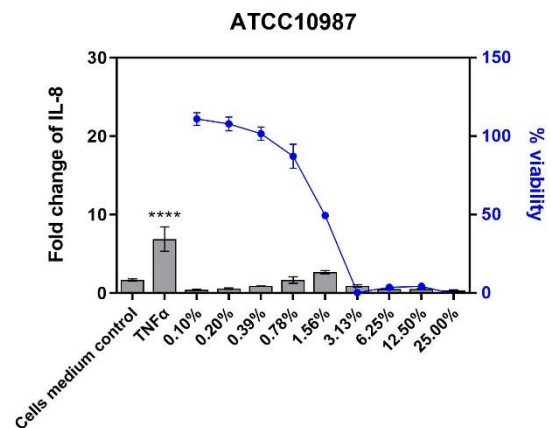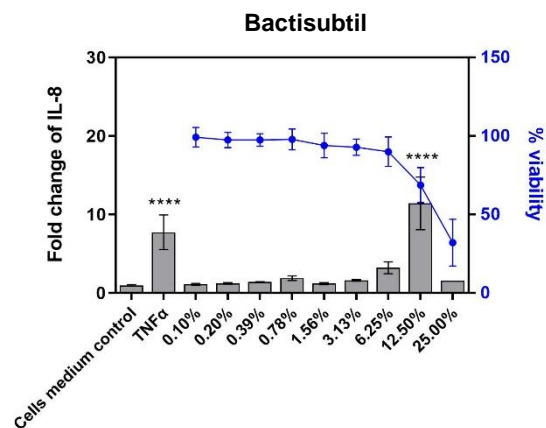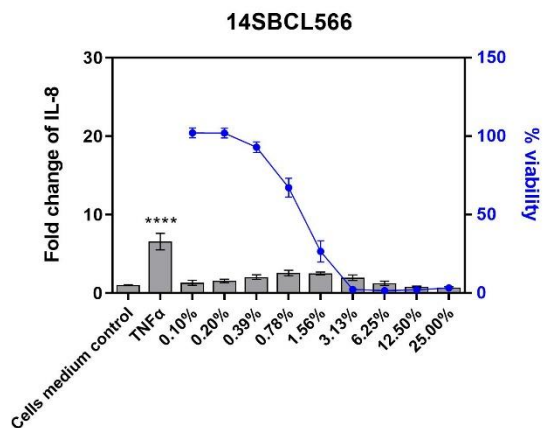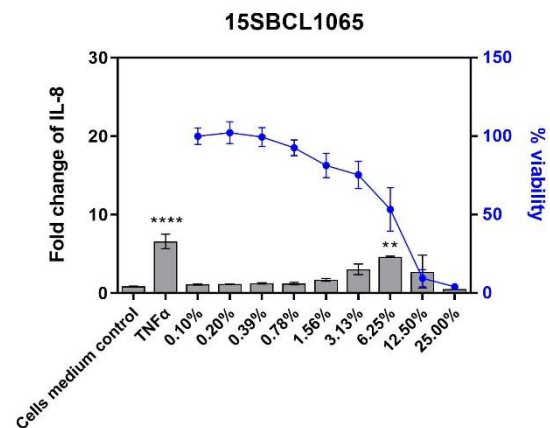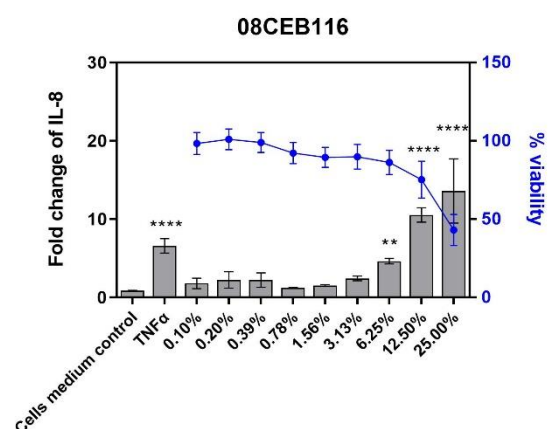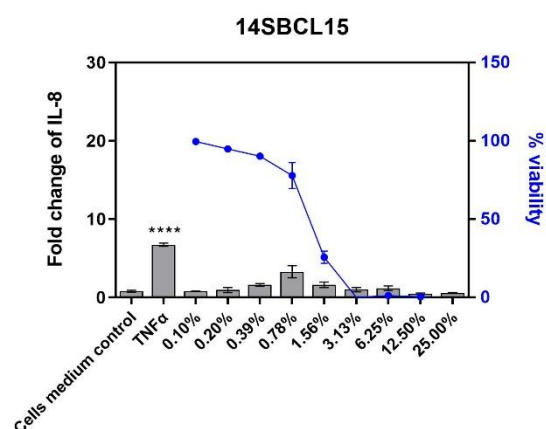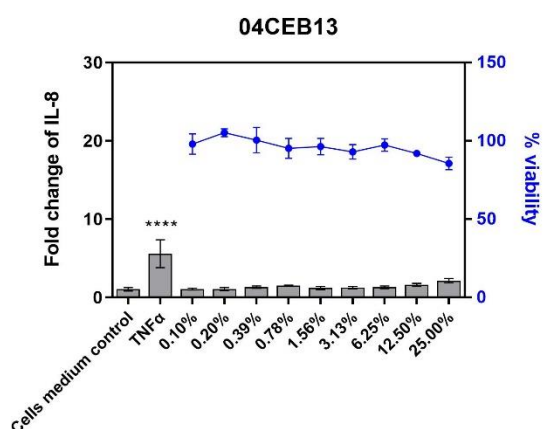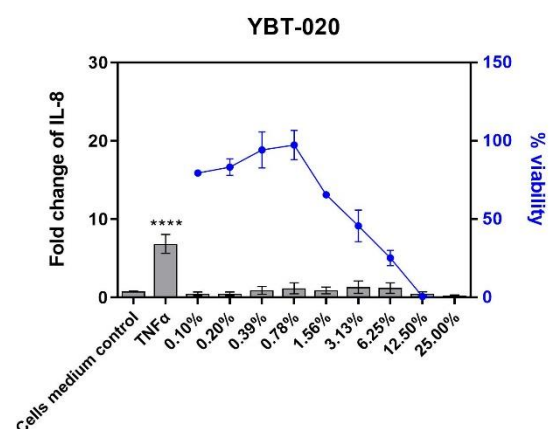

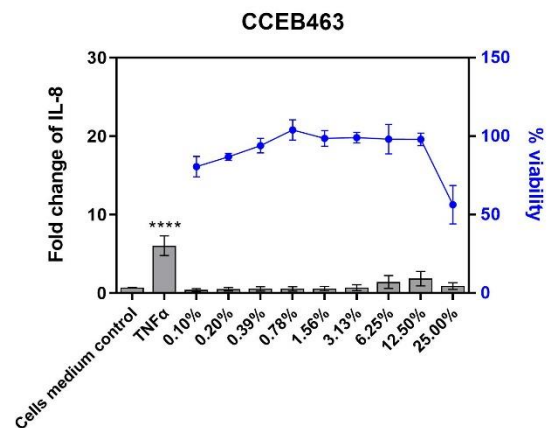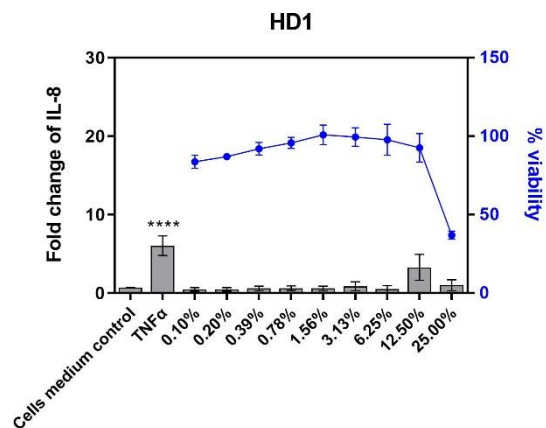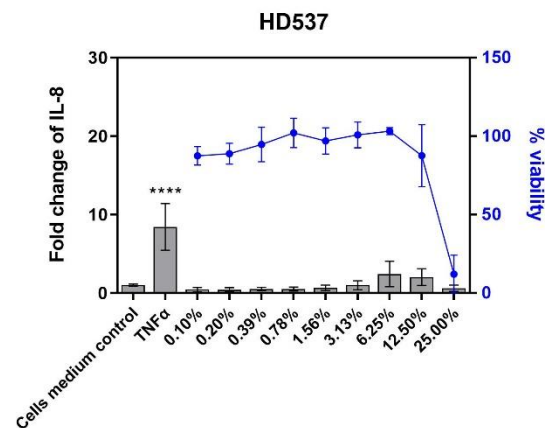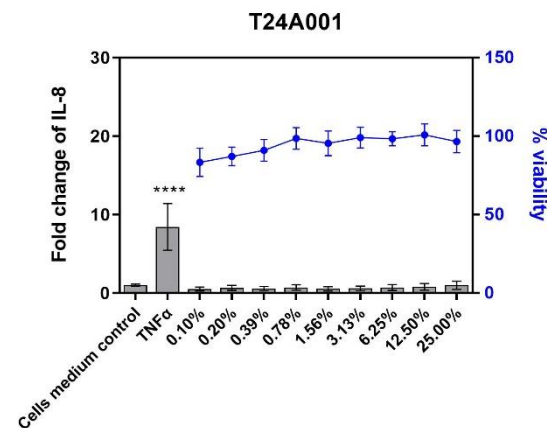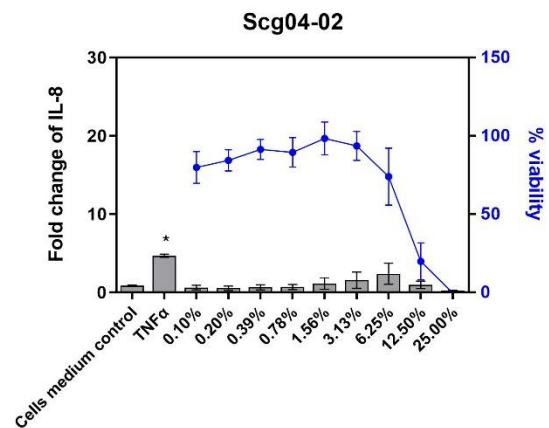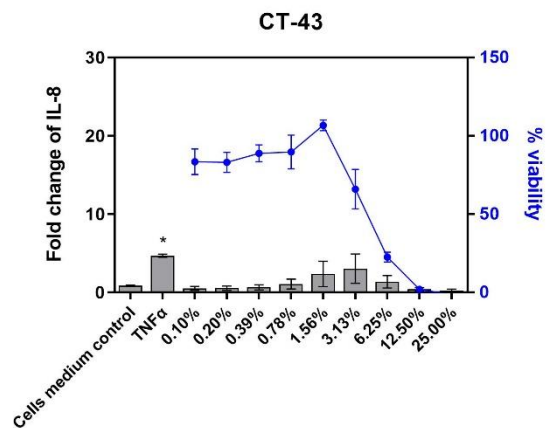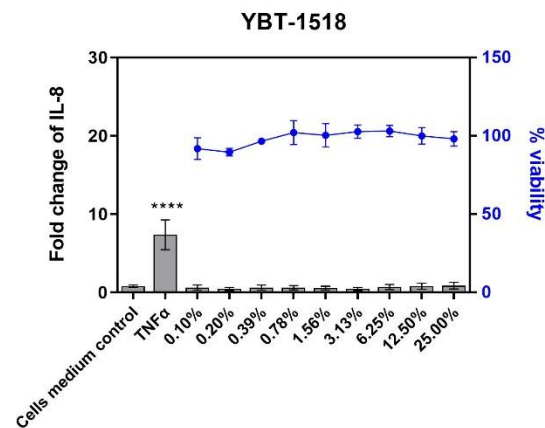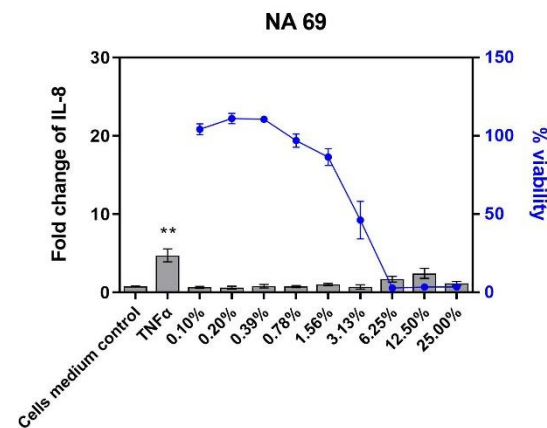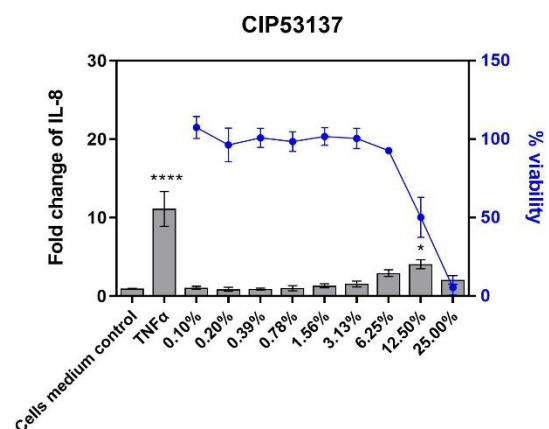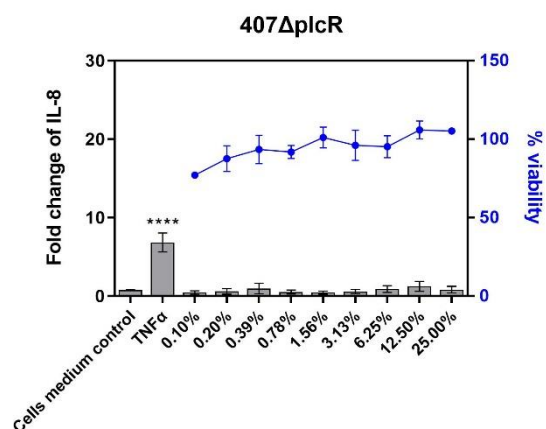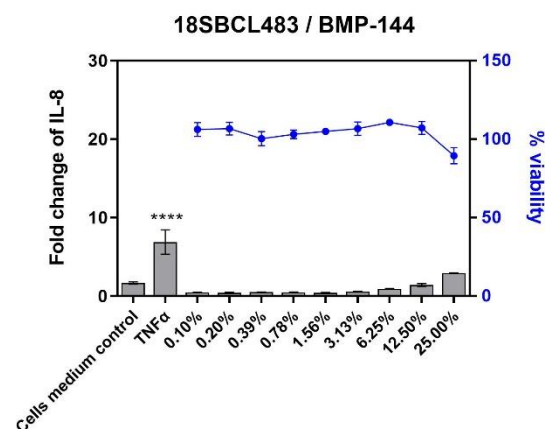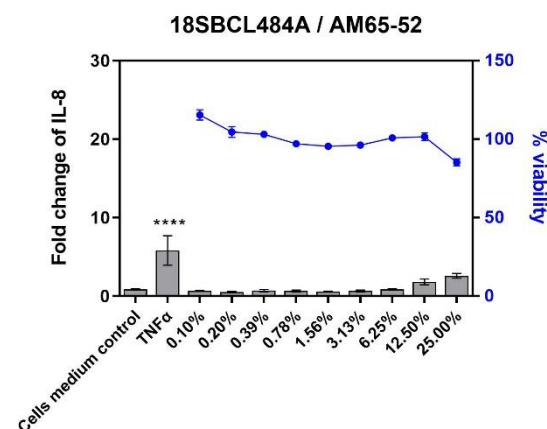



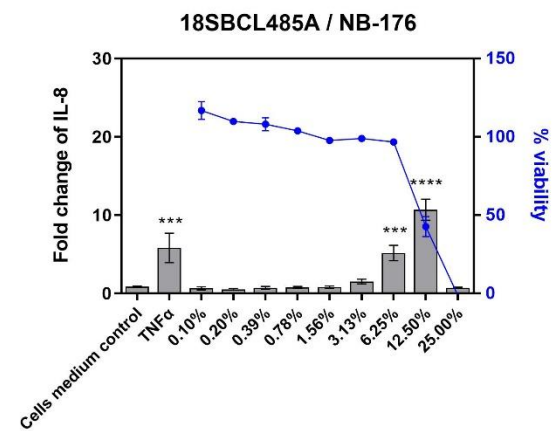

Supplement: Supplementary file 1 [file foods-13-01140-s001.zip › Figure S1.pdf]
